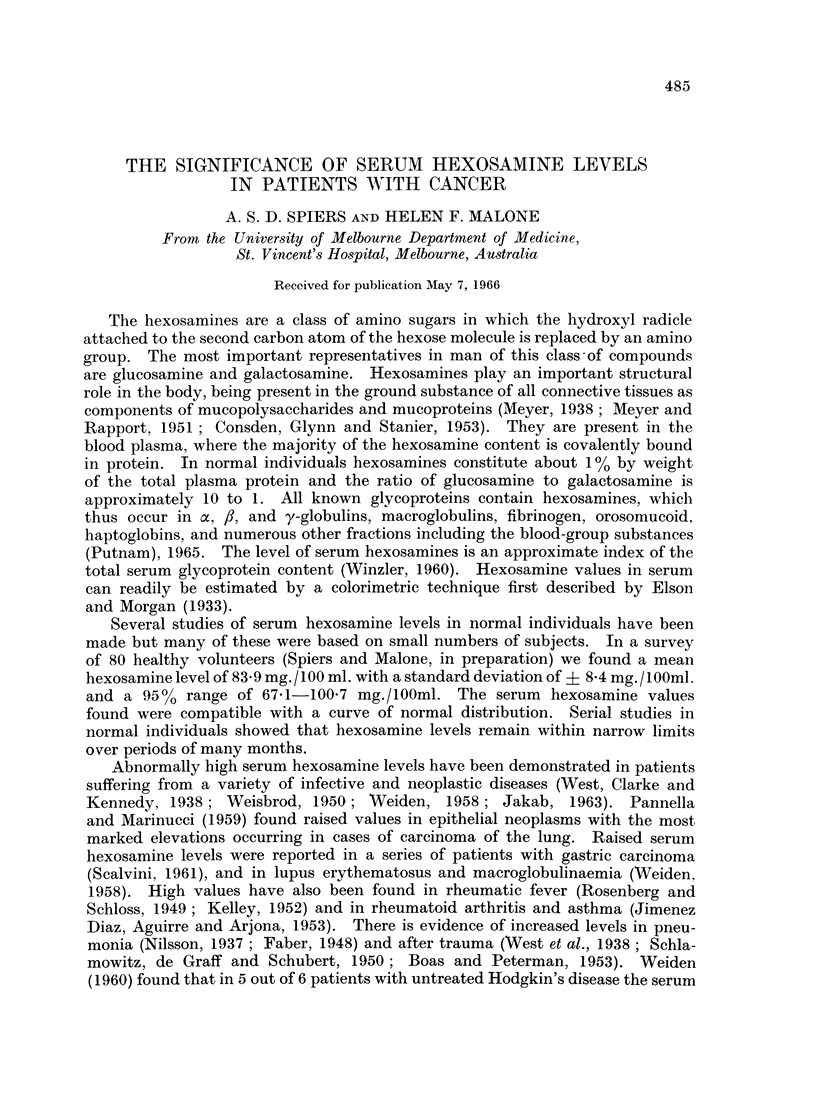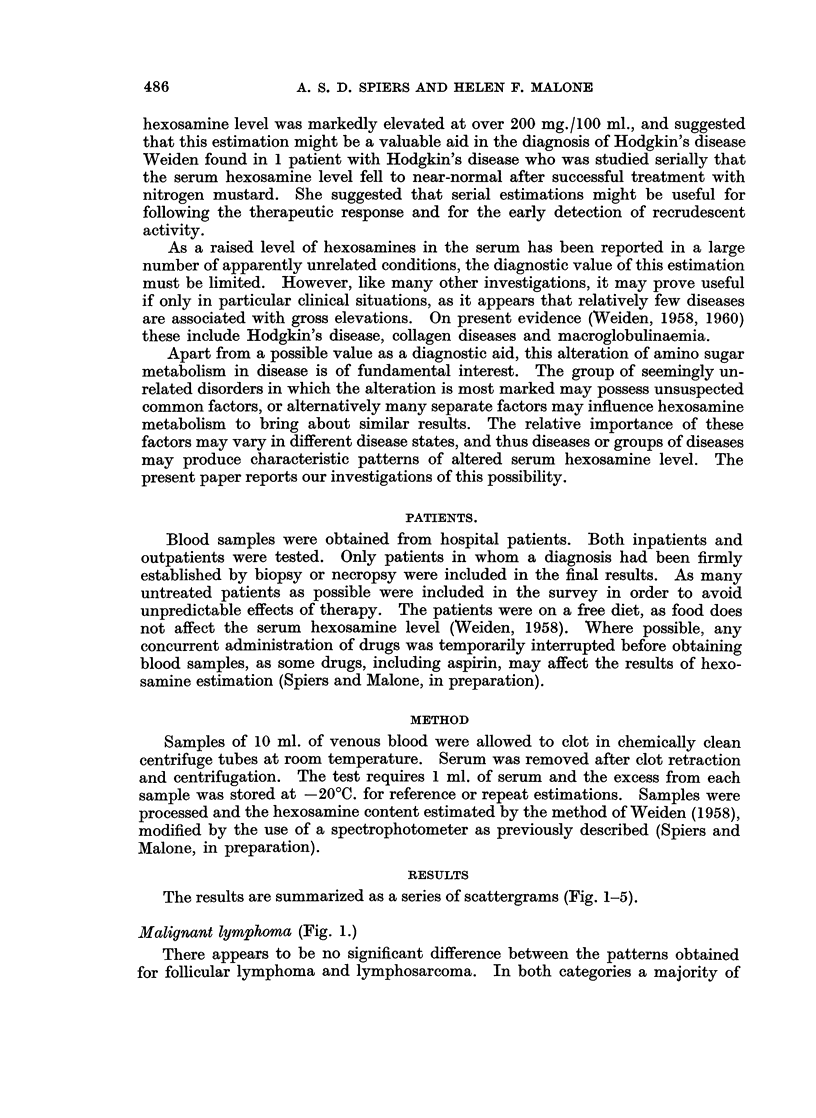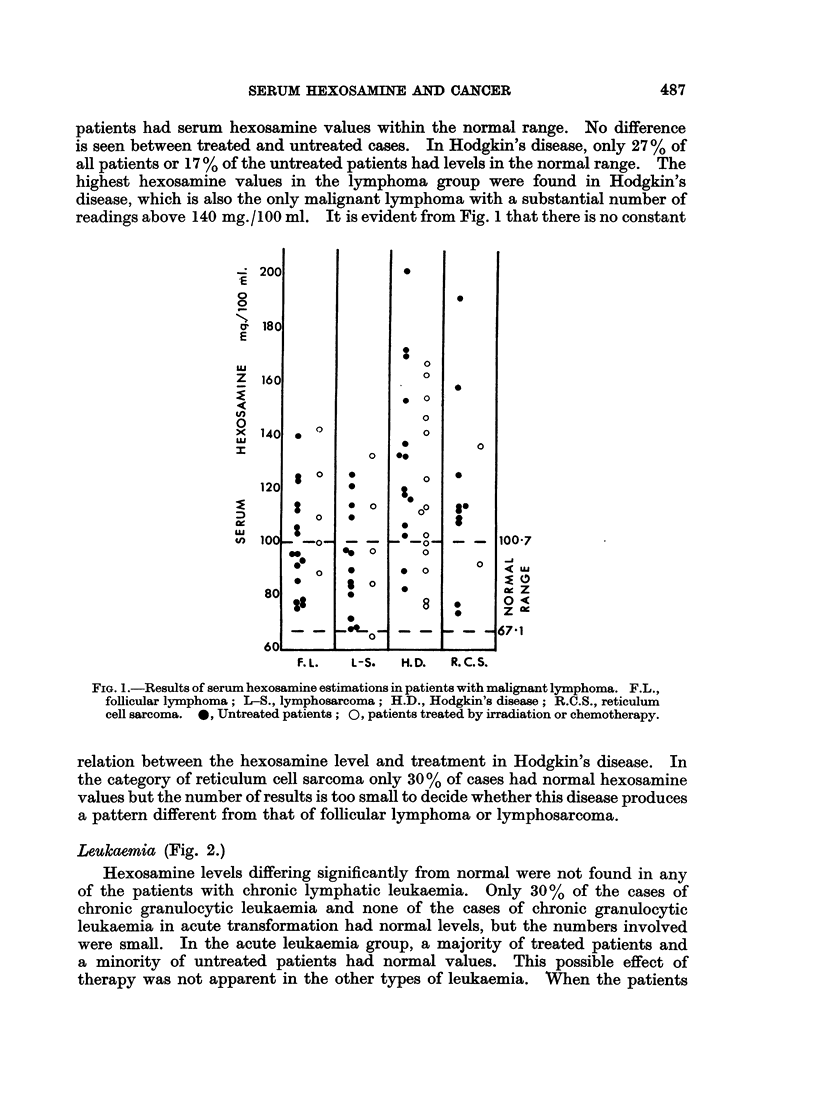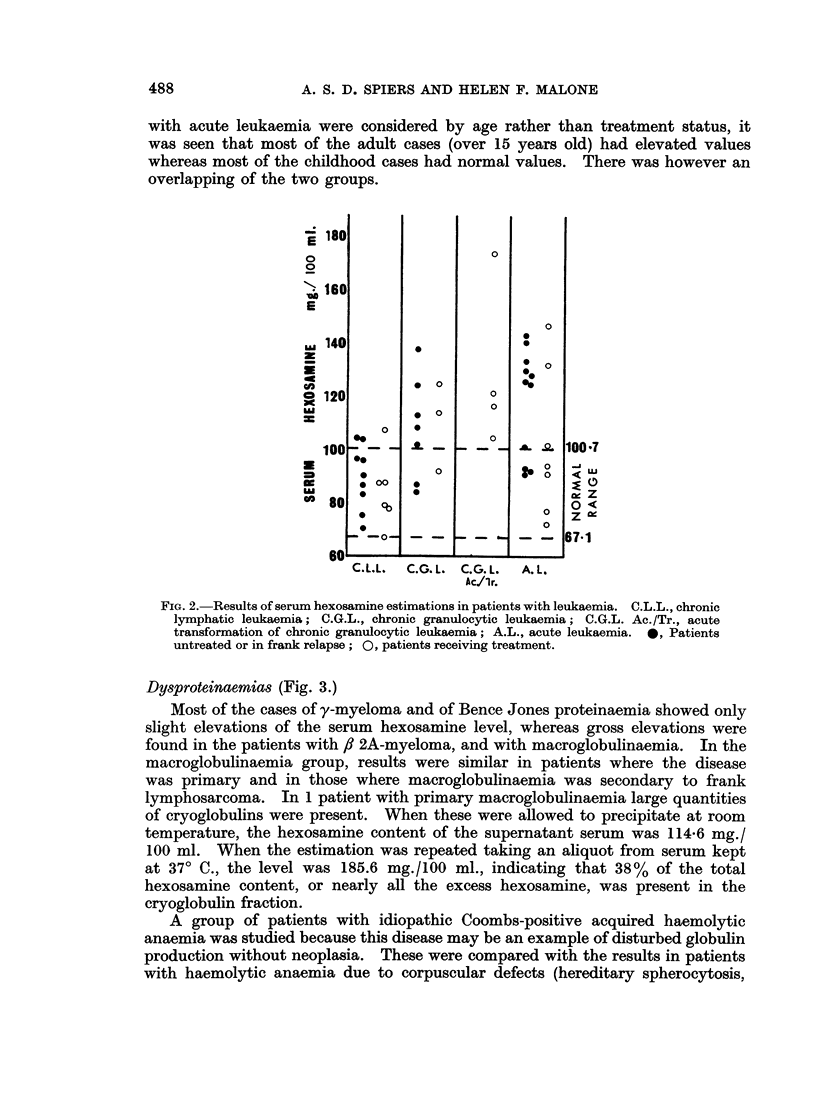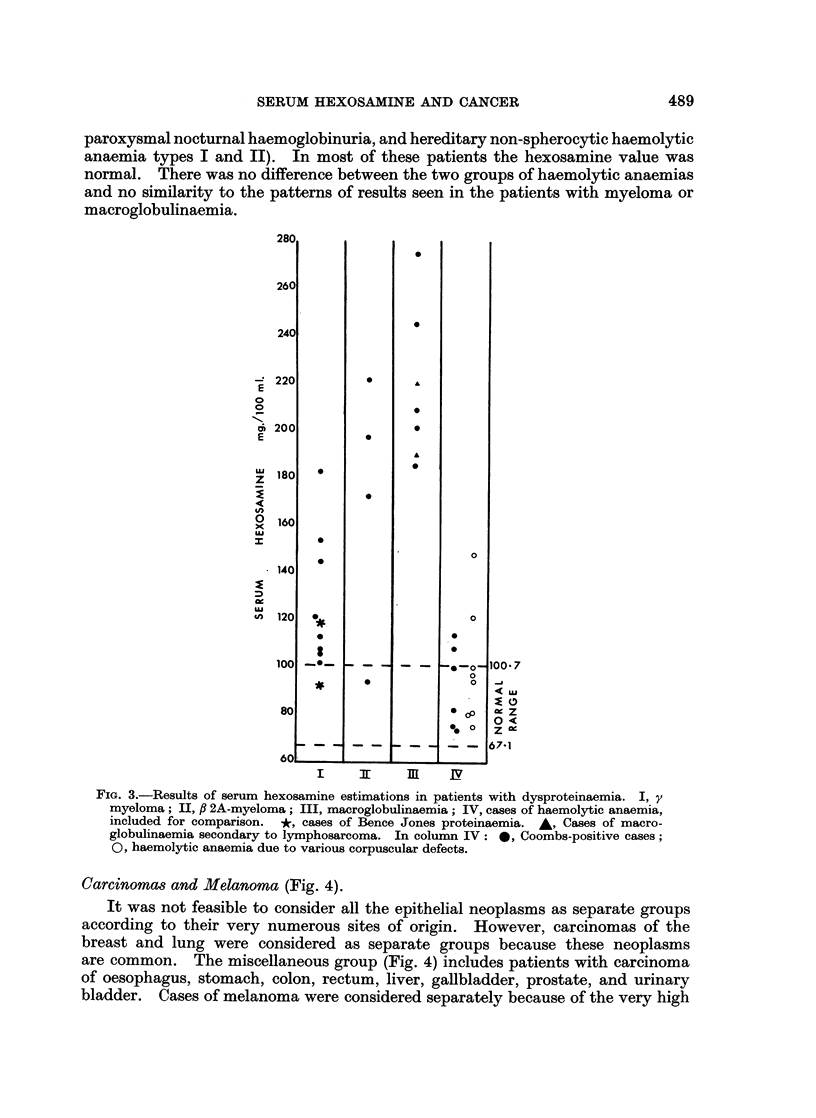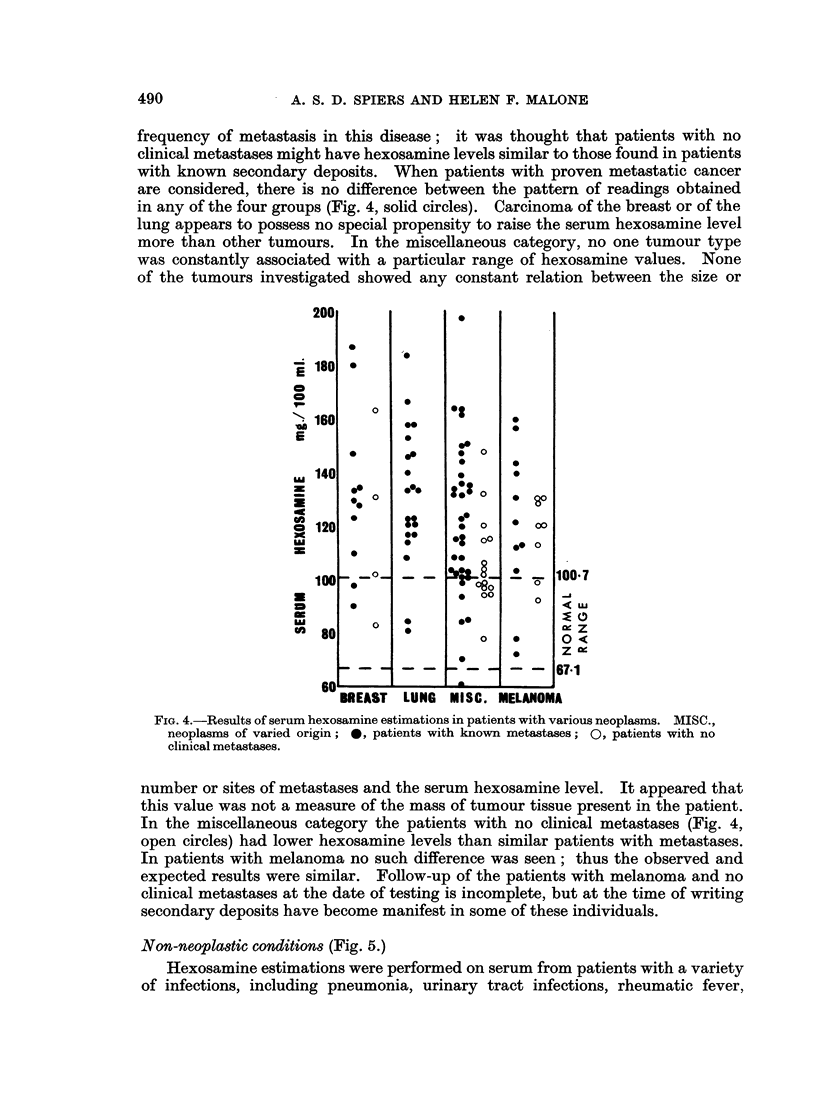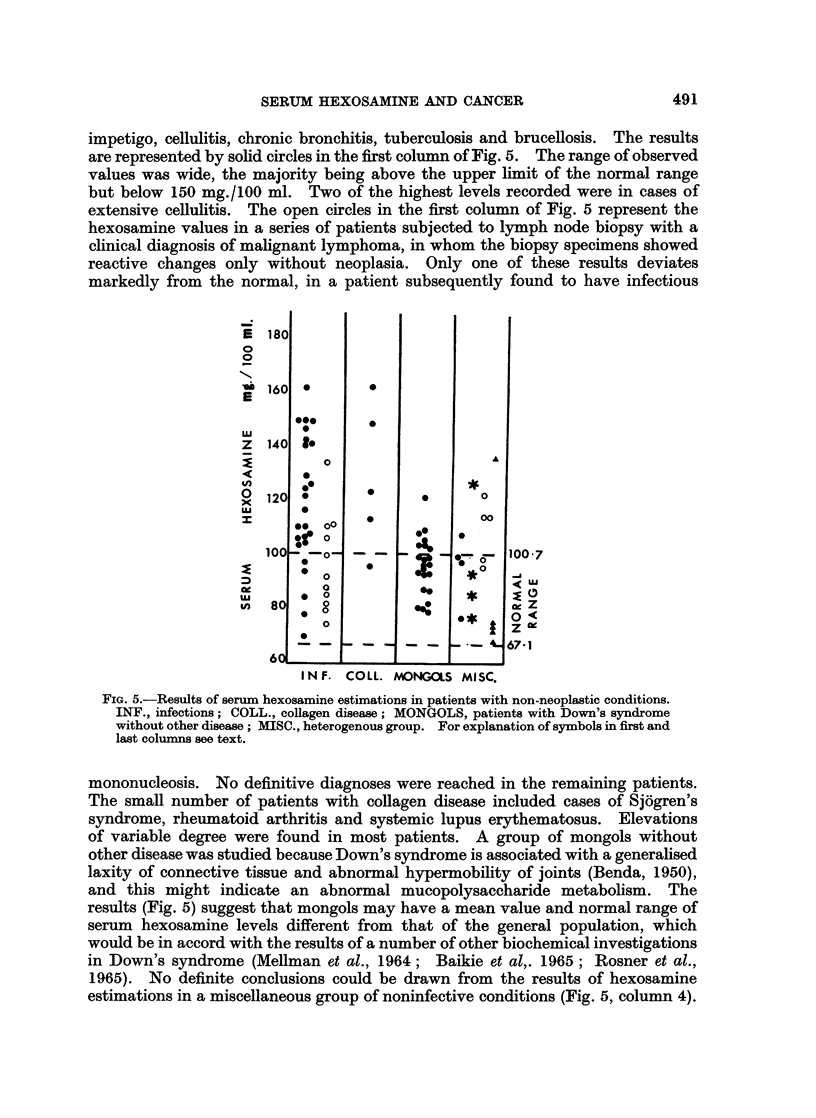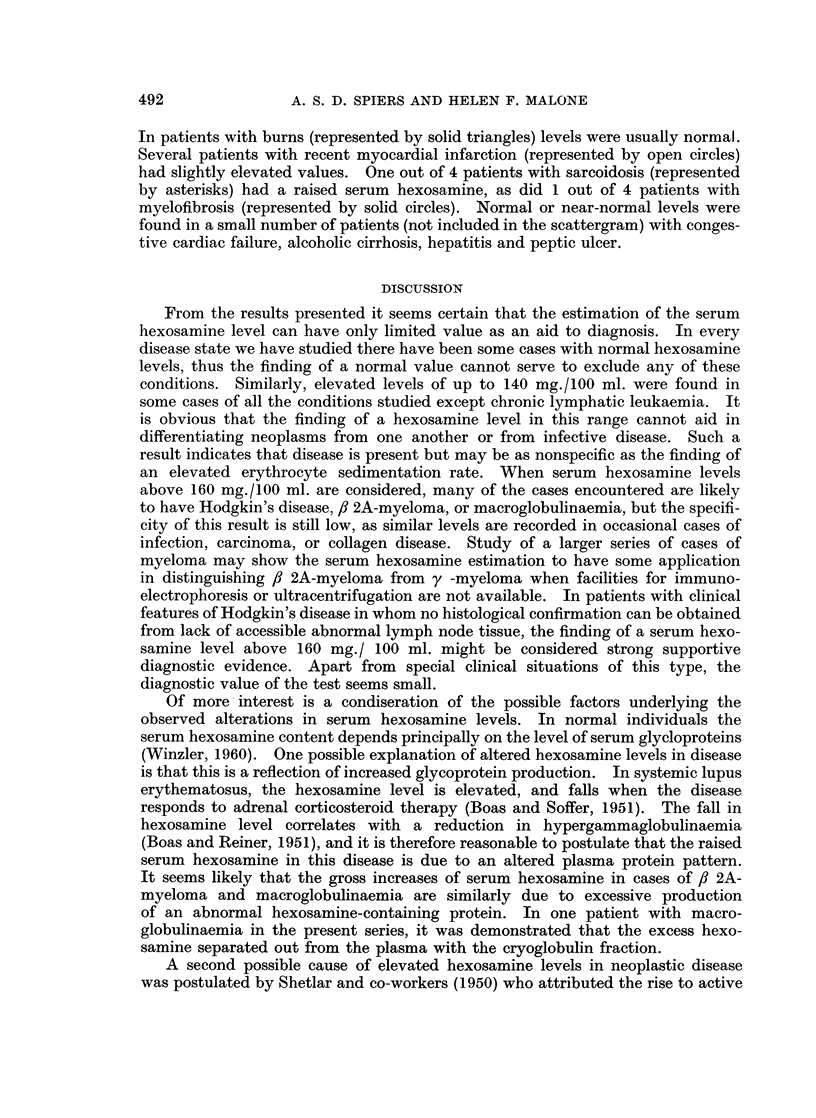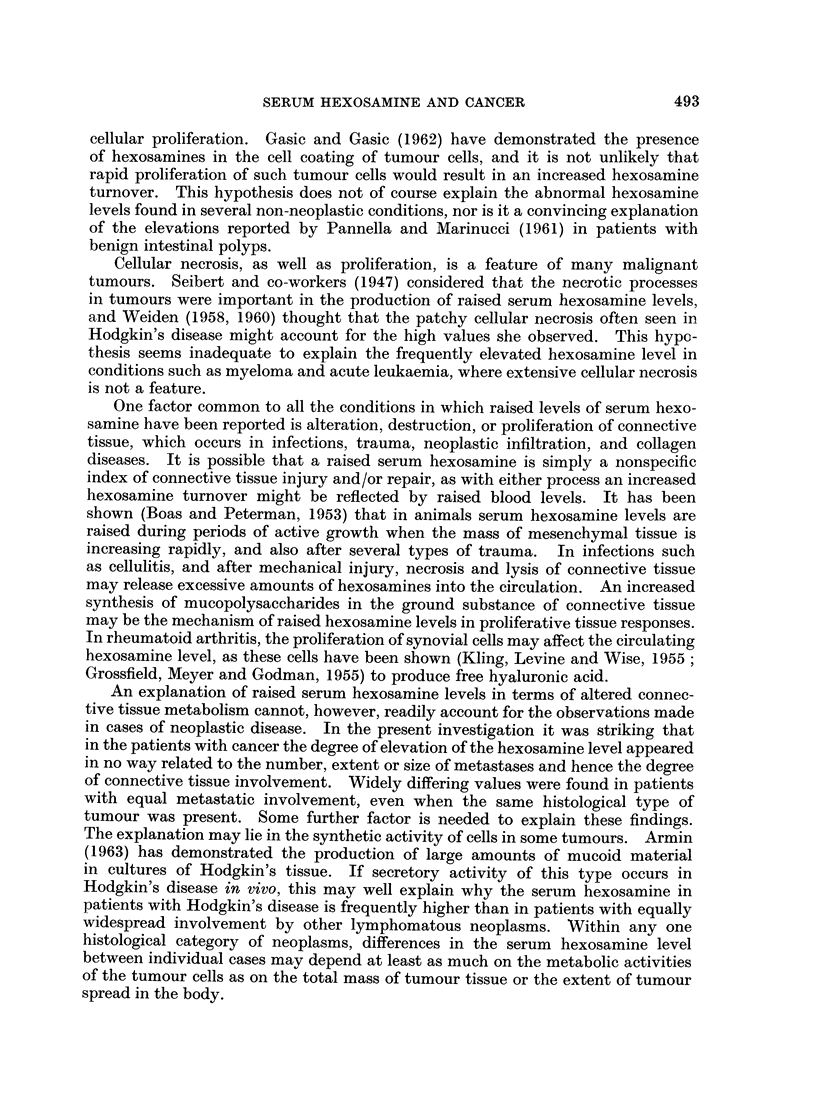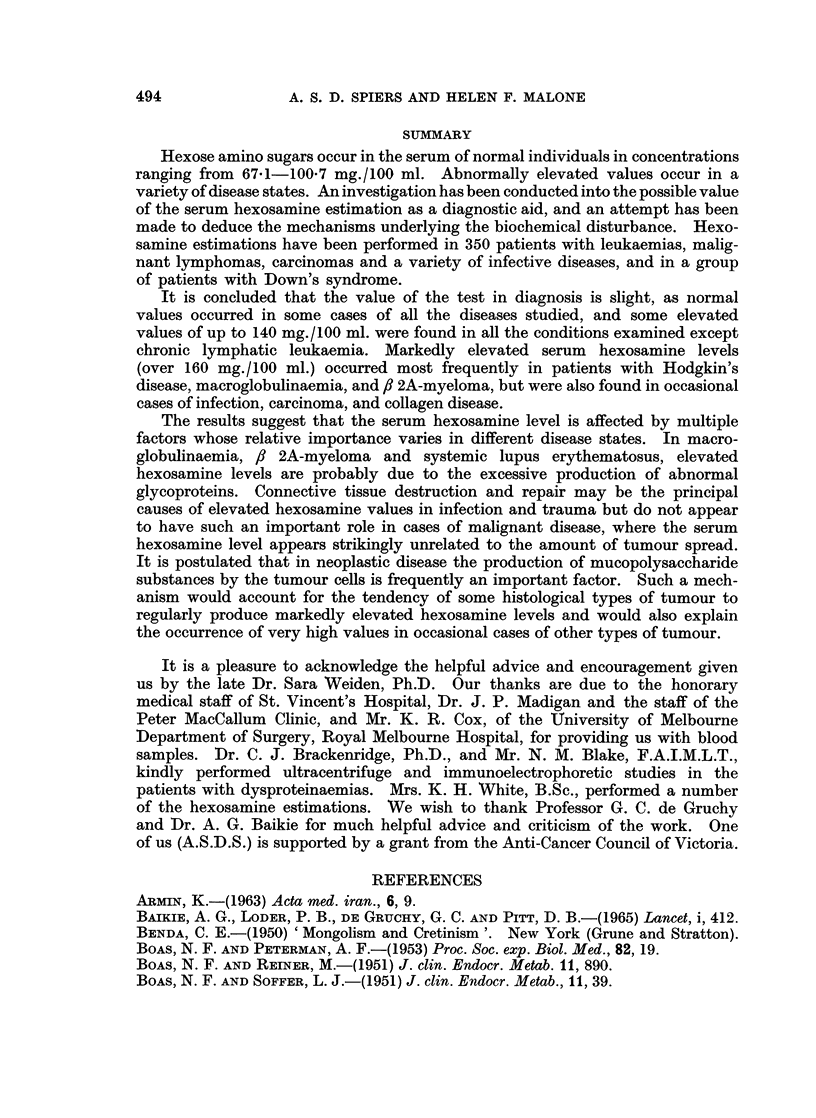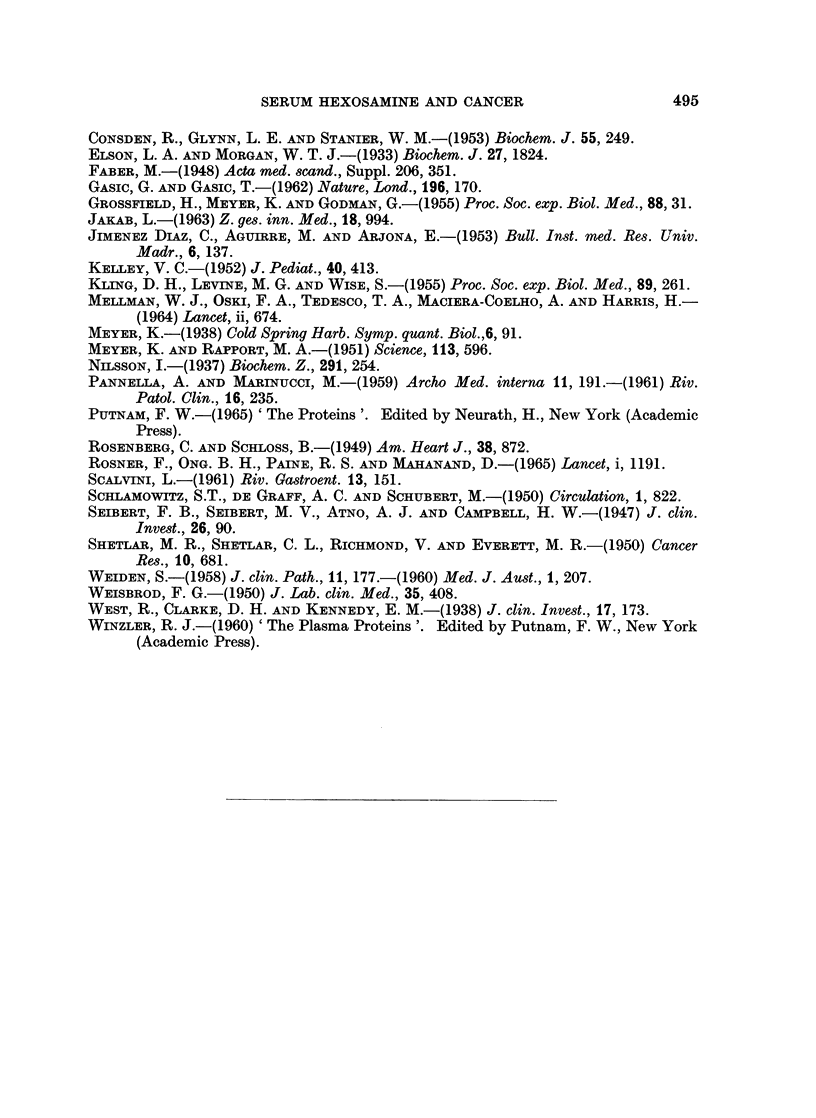# The significance of serum hexosamine levels in patients with cancer.

**DOI:** 10.1038/bjc.1966.60

**Published:** 1966-09

**Authors:** A. S. Spiers, H. F. Malone


### THE SIGNIFICANCE OF SERUM HEXOSAMINE LEVELS IN PATIENTS WITH CANCER

A. S. D. SPIERS AND HELEN F. MALONE

*From the University of Melbourne Department of Medicine, St. Vincent's Hospital, Melbourne, Australia*

Received for publication May 7, 1966

The hexosamines are a class of amino sugars in which the hydroxyl radicle attached to the second carbon atom of the hexose molecule is replaced by an amino group. The most important representatives in man of this class of compounds are glucosamine and galactosamine. Hexosamines play an important structural role in the body, being present in the ground substance of all connective tissues as components of mucopolysaccharides and mucoproteins (Meyer, 1938 ; Meyer and Rapport, 1951 ; Consden, Glynn and Stanier, 1953). They are present in the blood plasma, where the majority of the hexosamine content is covalently bound in protein. In normal individuals hexosamines constitute about 1% by weight of the total plasma protein and the ratio of glucosamine to galactosamine is approximately 10 to 1. All known glycoproteins contain hexosamines, which thus occur in $\alpha$, $\beta$, and $\gamma$-globulins, macroglobulins, fibrinogen, orosomucoid, haptoglobins, and numerous other fractions including the blood-group substances (Putnam), 1965. The level of serum hexosamines is an approximate index of the total serum glycoprotein content (Winzler, 1960). Hexosamine values in serum can readily be estimated by a colorimetric technique first described by Elson and Morgan (1933).

Several studies of serum hexosamine levels in normal individuals have been made but many of these were based on small numbers of subjects. In a survey of 80 healthy volunteers (Spiers and Malone, in preparation) we found a mean hexosamine level of 83·9 mg./100 ml. with a standard deviation of $\pm$ 8·4 mg./100ml. and a 95% range of 67·1—100·7 mg./100ml. The serum hexosamine values found were compatible with a curve of normal distribution. Serial studies in normal individuals showed that hexosamine levels remain within narrow limits over periods of many months.

Abnormally high serum hexosamine levels have been demonstrated in patients suffering from a variety of infective and neoplastic diseases (West, Clarke and Kennedy, 1938 ; Weisbrod, 1950 ; Weiden, 1958 ; Jakab, 1963). Pannella and Marinucci (1959) found raised values in epithelial neoplasms with the most marked elevations occurring in cases of carcinoma of the lung. Raised serum hexosamine levels were reported in a series of patients with gastric carcinoma (Scalvini, 1961), and in lupus erythematosus and macroglobulinaemia (Weiden, 1958). High values have also been found in rheumatic fever (Rosenberg and Schloss, 1949 ; Kelley, 1952) and in rheumatoid arthritis and asthma (Jimenez Diaz, Aguirre and Arjona, 1953). There is evidence of increased levels in pneumonia (Nilsson, 1937 ; Faber, 1948) and after trauma (West *et al.*, 1938 ; Schlamowitz, de Graff and Schubert, 1950 ; Boas and Peterman, 1953). Weiden (1960) found that in 5 out of 6 patients with untreated Hodgkin's disease the serum hexosamine level was markedly elevated at over 200 mg./100 ml., and suggested that this estimation might be a valuable aid in the diagnosis of Hodgkin's disease Weiden found in 1 patient with Hodgkin's disease who was studied serially that the serum hexosamine level fell to near-normal after successful treatment with nitrogen mustard. She suggested that serial estimations might be useful for following the therapeutic response and for the early detection of recrudescent activity.

As a raised level of hexosamines in the serum has been reported in a large number of apparently unrelated conditions, the diagnostic value of this estimation must be limited. However, like many other investigations, it may prove useful if only in particular clinical situations, as it appears that relatively few diseases are associated with gross elevations. On present evidence (Weiden, 1958, 1960) these include Hodgkin's disease, collagen diseases and macroglobulinaemia.

Apart from a possible value as a diagnostic aid, this alteration of amino sugar metabolism in disease is of fundamental interest. The group of seemingly unrelated disorders in which the alteration is most marked may possess unsuspected common factors, or alternatively many separate factors may influence hexosamine metabolism to bring about similar results. The relative importance of these factors may vary in different disease states, and thus diseases or groups of diseases may produce characteristic patterns of altered serum hexosamine level. The present paper reports our investigations of this possibility.

### PATIENTS.

Blood samples were obtained from hospital patients. Both inpatients and outpatients were tested. Only patients in whom a diagnosis had been firmly established by biopsy or necropsy were included in the final results. As many untreated patients as possible were included in the survey in order to avoid unpredictable effects of therapy. The patients were on a free diet, as food does not affect the serum hexosamine level (Weiden, 1958). Where possible, any concurrent administration of drugs was temporarily interrupted before obtaining blood samples, as some drugs, including aspirin, may affect the results of hexosamine estimation (Spiers and Malone, in preparation).

### METHOD

Samples of 10 ml. of venous blood were allowed to clot in chemically clean centrifuge tubes at room temperature. Serum was removed after clot retraction and centrifugation. The test requires 1 ml. of serum and the excess from each sample was stored at −20°C. for reference or repeat estimations. Samples were processed and the hexosamine content estimated by the method of Weiden (1958), modified by the use of a spectrophotometer as previously described (Spiers and Malone, in preparation).

### RESULTS

The results are summarized as a series of scattergrams (Fig. 1–5).

### *Malignant lymphoma* (Fig. 1.)

There appears to be no significant difference between the patterns obtained for follicular lymphoma and lymphosarcoma. In both categories a majority of patients had serum hexosamine values within the normal range. No difference is seen between treated and untreated cases. In Hodgkin's disease, only 27% of all patients or 17% of the untreated patients had levels in the normal range. The highest hexosamine values in the lymphoma group were found in Hodgkin's disease, which is also the only malignant lymphoma with a substantial number of readings above 140 mg./100 ml. It is evident from Fig. 1 that there is no constant

FIG. 1.—Results of serum hexosamine estimations in patients with malignant lymphoma. F.L., follicular lymphoma ; L–S., lymphosarcoma ; H.D., Hodgkin's disease ; R.C.S., reticulum cell sarcoma. ●, Untreated patients ; ○, patients treated by irradiation or chemotherapy.

relation between the hexosamine level and treatment in Hodgkin's disease. In the category of reticulum cell sarcoma only 30% of cases had normal hexosamine values but the number of results is too small to decide whether this disease produces a pattern different from that of follicular lymphoma or lymphosarcoma.

*Leukaemia* (Fig. 2.)

Hexosamine levels differing significantly from normal were not found in any of the patients with chronic lymphatic leukaemia. Only 30% of the cases of chronic granulocytic leukaemia and none of the cases of chronic granulocytic leukaemia in acute transformation had normal levels, but the numbers involved were small. In the acute leukaemia group, a majority of treated patients and a minority of untreated patients had normal values. This possible effect of therapy was not apparent in the other types of leukaemia. When the patients with acute leukaemia were considered by age rather than treatment status, it was seen that most of the adult cases (over 15 years old) had elevated values whereas most of the childhood cases had normal values. There was however an overlapping of the two groups.

FIG. 2.—Results of serum hexosamine estimations in patients with leukaemia. C.L.L., chronic lymphatic leukaemia; C.G.L., chronic granulocytic leukaemia; C.G.L. Ac./Tr., acute transformation of chronic granulocytic leukaemia; A.L., acute leukaemia. ●, Patients untreated or in frank relapse; ○, patients receiving treatment.

*Dysproteinaemias* (Fig. 3.)

Most of the cases of $\gamma$-myeloma and of Bence Jones proteinaemia showed only slight elevations of the serum hexosamine level, whereas gross elevations were found in the patients with $\beta$ 2A-myeloma, and with macroglobulinaemia. In the macroglobulinaemia group, results were similar in patients where the disease was primary and in those where macroglobulinaemia was secondary to frank lymphosarcoma. In 1 patient with primary macroglobulinaemia large quantities of cryoglobulins were present. When these were allowed to precipitate at room temperature, the hexosamine content of the supernatant serum was 114·6 mg./100 ml. When the estimation was repeated taking an aliquot from serum kept at 37° C., the level was 185.6 mg./100 ml., indicating that 38% of the total hexosamine content, or nearly all the excess hexosamine, was present in the cryoglobulin fraction.

A group of patients with idiopathic Coombs-positive acquired haemolytic anaemia was studied because this disease may be an example of disturbed globulin production without neoplasia. These were compared with the results in patients with haemolytic anaemia due to corpuscular defects (hereditary spherocytosis, paroxysmal nocturnal haemoglobinuria, and hereditary non-spherocytic haemolytic anaemia types I and II). In most of these patients the hexosamine value was normal. There was no difference between the two groups of haemolytic anaemias and no similarity to the patterns of results seen in the patients with myeloma or macroglobulinaemia.

FIG. 3.—Results of serum hexosamine estimations in patients with dysproteinaemia. I, $\gamma$ myeloma ; II, $\beta$ 2A-myeloma ; III, macroglobulinaemia ; IV, cases of haemolytic anaemia, included for comparison. ★, cases of Bence Jones proteinaemia. ▲, Cases of macroglobulinaemia secondary to lymphosarcoma. In column IV : ●, Coombs-positive cases ; ○, haemolytic anaemia due to various corpuscular defects.

*Carcinomas and Melanoma* (Fig. 4).

It was not feasible to consider all the epithelial neoplasms as separate groups according to their very numerous sites of origin. However, carcinomas of the breast and lung were considered as separate groups because these neoplasms are common. The miscellaneous group (Fig. 4) includes patients with carcinoma of oesophagus, stomach, colon, rectum, liver, gallbladder, prostate, and urinary bladder. Cases of melanoma were considered separately because of the very high frequency of metastasis in this disease ; it was thought that patients with no clinical metastases might have hexosamine levels similar to those found in patients with known secondary deposits. When patients with proven metastatic cancer are considered, there is no difference between the pattern of readings obtained in any of the four groups (Fig. 4, solid circles). Carcinoma of the breast or of the lung appears to possess no special propensity to raise the serum hexosamine level more than other tumours. In the miscellaneous category, no one tumour type was constantly associated with a particular range of hexosamine values. None of the tumours investigated showed any constant relation between the size or

Fig. 4.—Results of serum hexosamine estimations in patients with various neoplasms. MISC., neoplasms of varied origin ; ●, patients with known metastases ; ○, patients with no clinical metastases.

number or sites of metastases and the serum hexosamine level. It appeared that this value was not a measure of the mass of tumour tissue present in the patient. In the miscellaneous category the patients with no clinical metastases (Fig. 4, open circles) had lower hexosamine levels than similar patients with metastases. In patients with melanoma no such difference was seen ; thus the observed and expected results were similar. Follow-up of the patients with melanoma and no clinical metastases at the date of testing is incomplete, but at the time of writing secondary deposits have become manifest in some of these individuals.

*Non-neoplastic conditions* (Fig. 5.)

Hexosamine estimations were performed on serum from patients with a variety of infections, including pneumonia, urinary tract infections, rheumatic fever, impetigo, cellulitis, chronic bronchitis, tuberculosis and brucellosis. The results are represented by solid circles in the first column of Fig. 5. The range of observed values was wide, the majority being above the upper limit of the normal range but below 150 mg./100 ml. Two of the highest levels recorded were in cases of extensive cellulitis. The open circles in the first column of Fig. 5 represent the hexosamine values in a series of patients subjected to lymph node biopsy with a clinical diagnosis of malignant lymphoma, in whom the biopsy specimens showed reactive changes only without neoplasia. Only one of these results deviates markedly from the normal, in a patient subsequently found to have infectious

FIG. 5.—Results of serum hexosamine estimations in patients with non-neoplastic conditions. INF., infections ; COLL., collagen disease ; MONGOLS, patients with Down's syndrome without other disease ; MISC., heterogenous group. For explanation of symbols in first and last columns see text.

mononucleosis. No definitive diagnoses were reached in the remaining patients. The small number of patients with collagen disease included cases of Sjögren's syndrome, rheumatoid arthritis and systemic lupus erythematosus. Elevations of variable degree were found in most patients. A group of mongols without other disease was studied because Down's syndrome is associated with a generalised laxity of connective tissue and abnormal hypermobility of joints (Benda, 1950), and this might indicate an abnormal mucopolysaccharide metabolism. The results (Fig. 5) suggest that mongols may have a mean value and normal range of serum hexosamine levels different from that of the general population, which would be in accord with the results of a number of other biochemical investigations in Down's syndrome (Mellman *et al.*, 1964 ; Baikie *et al*,. 1965 ; Rosner *et al.*, 1965). No definite conclusions could be drawn from the results of hexosamine estimations in a miscellaneous group of noninfective conditions (Fig. 5, column 4).

In patients with burns (represented by solid triangles) levels were usually normal. Several patients with recent myocardial infarction (represented by open circles) had slightly elevated values. One out of 4 patients with sarcoidosis (represented by asterisks) had a raised serum hexosamine, as did 1 out of 4 patients with myelofibrosis (represented by solid circles). Normal or near-normal levels were found in a small number of patients (not included in the scattergram) with congestive cardiac failure, alcoholic cirrhosis, hepatitis and peptic ulcer.

### DISCUSSION

From the results presented it seems certain that the estimation of the serum hexosamine level can have only limited value as an aid to diagnosis. In every disease state we have studied there have been some cases with normal hexosamine levels, thus the finding of a normal value cannot serve to exclude any of these conditions. Similarly, elevated levels of up to 140 mg./100 ml. were found in some cases of all the conditions studied except chronic lymphatic leukaemia. It is obvious that the finding of a hexosamine level in this range cannot aid in differentiating neoplasms from one another or from infective disease. Such a result indicates that disease is present but may be as nonspecific as the finding of an elevated erythrocyte sedimentation rate. When serum hexosamine levels above 160 mg./100 ml. are considered, many of the cases encountered are likely to have Hodgkin's disease, $\beta$ 2A-myeloma, or macroglobulinaemia, but the specificity of this result is still low, as similar levels are recorded in occasional cases of infection, carcinoma, or collagen disease. Study of a larger series of cases of myeloma may show the serum hexosamine estimation to have some application in distinguishing $\beta$ 2A-myeloma from $\gamma$ -myeloma when facilities for immunoelectrophoresis or ultracentrifugation are not available. In patients with clinical features of Hodgkin's disease in whom no histological confirmation can be obtained from lack of accessible abnormal lymph node tissue, the finding of a serum hexosamine level above 160 mg./ 100 ml. might be considered strong supportive diagnostic evidence. Apart from special clinical situations of this type, the diagnostic value of the test seems small.

Of more interest is a condiseration of the possible factors underlying the observed alterations in serum hexosamine levels. In normal individuals the serum hexosamine content depends principally on the level of serum glycloproteins (Winzler, 1960). One possible explanation of altered hexosamine levels in disease is that this is a reflection of increased glycoprotein production. In systemic lupus erythematosus, the hexosamine level is elevated, and falls when the disease responds to adrenal corticosteroid therapy (Boas and Soffer, 1951). The fall in hexosamine level correlates with a reduction in hypergammaglobulinaemia (Boas and Reiner, 1951), and it is therefore reasonable to postulate that the raised serum hexosamine in this disease is due to an altered plasma protein pattern. It seems likely that the gross increases of serum hexosamine in cases of $\beta$ 2A-myeloma and macroglobulinaemia are similarly due to excessive production of an abnormal hexosamine-containing protein. In one patient with macroglobulinaemia in the present series, it was demonstrated that the excess hexosamine separated out from the plasma with the cryoglobulin fraction.

A second possible cause of elevated hexosamine levels in neoplastic disease was postulated by Shetlar and co-workers (1950) who attributed the rise to active cellular proliferation. Gasic and Gasic (1962) have demonstrated the presence of hexosamines in the cell coating of tumour cells, and it is not unlikely that rapid proliferation of such tumour cells would result in an increased hexosamine turnover. This hypothesis does not of course explain the abnormal hexosamine levels found in several non-neoplastic conditions, nor is it a convincing explanation of the elevations reported by Pannella and Marinucci (1961) in patients with benign intestinal polyps.

Cellular necrosis, as well as proliferation, is a feature of many malignant tumours. Seibert and co-workers (1947) considered that the necrotic processes in tumours were important in the production of raised serum hexosamine levels, and Weiden (1958, 1960) thought that the patchy cellular necrosis often seen in Hodgkin's disease might account for the high values she observed. This hypothesis seems inadequate to explain the frequently elevated hexosamine level in conditions such as myeloma and acute leukaemia, where extensive cellular necrosis is not a feature.

One factor common to all the conditions in which raised levels of serum hexosamine have been reported is alteration, destruction, or proliferation of connective tissue, which occurs in infections, trauma, neoplastic infiltration, and collagen diseases. It is possible that a raised serum hexosamine is simply a nonspecific index of connective tissue injury and/or repair, as with either process an increased hexosamine turnover might be reflected by raised blood levels. It has been shown (Boas and Peterman, 1953) that in animals serum hexosamine levels are raised during periods of active growth when the mass of mesenchymal tissue is increasing rapidly, and also after several types of trauma. In infections such as cellulitis, and after mechanical injury, necrosis and lysis of connective tissue may release excessive amounts of hexosamines into the circulation. An increased synthesis of mucopolysaccharides in the ground substance of connective tissue may be the mechanism of raised hexosamine levels in proliferative tissue responses. In rheumatoid arthritis, the proliferation of synovial cells may affect the circulating hexosamine level, as these cells have been shown (Kling, Levine and Wise, 1955 ; Grossfield, Meyer and Godman, 1955) to produce free hyaluronic acid.

An explanation of raised serum hexosamine levels in terms of altered connective tissue metabolism cannot, however, readily account for the observations made in cases of neoplastic disease. In the present investigation it was striking that in the patients with cancer the degree of elevation of the hexosamine level appeared in no way related to the number, extent or size of metastases and hence the degree of connective tissue involvement. Widely differing values were found in patients with equal metastatic involvement, even when the same histological type of tumour was present. Some further factor is needed to explain these findings. The explanation may lie in the synthetic activity of cells in some tumours. Armin (1963) has demonstrated the production of large amounts of mucoid material in cultures of Hodgkin's tissue. If secretory activity of this type occurs in Hodgkin's disease *in vivo*, this may well explain why the serum hexosamine in patients with Hodgkin's disease is frequently higher than in patients with equally widespread involvement by other lymphomatous neoplasms. Within any one histological category of neoplasms, differences in the serum hexosamine level between individual cases may depend at least as much on the metabolic activities of the tumour cells as on the total mass of tumour tissue or the extent of tumour spread in the body.

SUMMARY

Hexose amino sugars occur in the serum of normal individuals in concentrations ranging from 67·1—100·7 mg./100 ml. Abnormally elevated values occur in a variety of disease states. An investigation has been conducted into the possible value of the serum hexosamine estimation as a diagnostic aid, and an attempt has been made to deduce the mechanisms underlying the biochemical disturbance. Hexosamine estimations have been performed in 350 patients with leukaemias, malignant lymphomas, carcinomas and a variety of infective diseases, and in a group of patients with Down's syndrome.

It is concluded that the value of the test in diagnosis is slight, as normal values occurred in some cases of all the diseases studied, and some elevated values of up to 140 mg./100 ml. were found in all the conditions examined except chronic lymphatic leukaemia. Markedly elevated serum hexosamine levels (over 160 mg./100 ml.) occurred most frequently in patients with Hodgkin's disease, macroglobulinaemia, and $\beta$ 2A-myeloma, but were also found in occasional cases of infection, carcinoma, and collagen disease.

The results suggest that the serum hexosamine level is affected by multiple factors whose relative importance varies in different disease states. In macroglobulinaemia, $\beta$ 2A-myeloma and systemic lupus erythematosus, elevated hexosamine levels are probably due to the excessive production of abnormal glycoproteins. Connective tissue destruction and repair may be the principal causes of elevated hexosamine values in infection and trauma but do not appear to have such an important role in cases of malignant disease, where the serum hexosamine level appears strikingly unrelated to the amount of tumour spread. It is postulated that in neoplastic disease the production of mucopolysaccharide substances by the tumour cells is frequently an important factor. Such a mechanism would account for the tendency of some histological types of tumour to regularly produce markedly elevated hexosamine levels and would also explain the occurrence of very high values in occasional cases of other types of tumour.

It is a pleasure to acknowledge the helpful advice and encouragement given us by the late Dr. Sara Weiden, Ph.D. Our thanks are due to the honorary medical staff of St. Vincent's Hospital, Dr. J. P. Madigan and the staff of the Peter MacCallum Clinic, and Mr. K. R. Cox, of the University of Melbourne Department of Surgery, Royal Melbourne Hospital, for providing us with blood samples. Dr. C. J. Brackenridge, Ph.D., and Mr. N. M. Blake, F.A.I.M.L.T., kindly performed ultracentrifuge and immunoelectrophoretic studies in the patients with dysproteinaemias. Mrs. K. H. White, B.Sc., performed a number of the hexosamine estimations. We wish to thank Professor G. C. de Gruchy and Dr. A. G. Baikie for much helpful advice and criticism of the work. One of us (A.S.D.S.) is supported by a grant from the Anti-Cancer Council of Victoria.

REFERENCES

ARMIN, K.—(1963) *Acta med. iran.*, **6**, 9.
BAIKIE, A. G., LODER, P. B., DE GRUCHY, G. C. AND PITT, D. B.—(1965) *Lancet*, i, 412.
BENDA, C. E.—(1950) ' Mongolism and Cretinism '. New York (Grune and Stratton).
BOAS, N. F. AND PETERMAN, A. F.—(1953) *Proc. Soc. exp. Biol. Med.*, **82**, 19.
BOAS, N. F. AND REINER, M.—(1951) *J. clin. Endocr. Metab.* **11**, 890.
BOAS, N. F. AND SOFFER, L. J.—(1951) *J. clin. Endocr. Metab.*, **11**, 39.

Consden, R., Glynn, L. E. and Stanier, W. M.—(1953) *Biochem. J.* **55**, 249.
Elson, L. A. and Morgan, W. T. J.—(1933) *Biochem. J.* **27**, 1824.
Faber, M.—(1948) *Acta med. scand.*, Suppl. 206, 351.
Gasic, G. and Gasic, T.—(1962) *Nature, Lond.*, **196**, 170.
Grossfield, H., Meyer, K. and Godman, G.—(1955) *Proc. Soc. exp. Biol. Med.*, **88**, 31.
Jakab, L.—(1963) *Z. ges. inn. Med.*, **18**, 994.
Jimenez Diaz, C., Aguirre, M. and Arjona, E.—(1953) *Bull. Inst. med. Res. Univ. Madr.*, **6**, 137.
Kelley, V. C.—(1952) *J. Pediat.*, **40**, 413.
Kling, D. H., Levine, M. G. and Wise, S.—(1955) *Proc. Soc. exp. Biol. Med.*, **89**, 261.
Mellman, W. J., Oski, F. A., Tedesco, T. A., Maciera-Coelho, A. and Harris, H.—(1964) *Lancet*, ii, 674.
Meyer, K.—(1938) *Cold Spring Harb. Symp. quant. Biol.*,**6**, 91.
Meyer, K. and Rapport, M. A.—(1951) *Science*, **113**, 596.
Nilsson, I.—(1937) *Biochem. Z.*, **291**, 254.
Pannella, A. and Marinucci, M.—(1959) *Archo Med. interna* **11**, 191.—(1961) *Riv. Patol. Clin.*, **16**, 235.
Putnam, F. W.—(1965) ' The Proteins '. Edited by Neurath, H., New York (Academic Press).
Rosenberg, C. and Schloss, B.—(1949) *Am. Heart J.*, **38**, 872.
Rosner, F., Ong. B. H., Paine, R. S. and Mahanand, D.—(1965) *Lancet*, i, 1191.
Scalvini, L.—(1961) *Riv. Gastroent.* **13**, 151.
Schlamowitz, S.T., de Graff, A. C. and Schubert, M.—(1950) *Circulation*, **1**, 822.
Seibert, F. B., Seibert, M. V., Atno, A. J. and Campbell, H. W.—(1947) *J. clin. Invest.*, **26**, 90.
Shetlar, M. R., Shetlar, C. L., Richmond, V. and Everett, M. R.—(1950) *Cancer Res.*, **10**, 681.
Weiden, S.—(1958) *J. clin. Path.*, **11**, 177.—(1960) *Med. J. Aust.*, **1**, 207.
Weisbrod, F. G.—(1950) *J. Lab. clin. Med.*, **35**, 408.
West, R., Clarke, D. H. and Kennedy, E. M.—(1938) *J. clin. Invest.*, **17**, 173.
Winzler, R. J.—(1960) ' The Plasma Proteins '. Edited by Putnam, F. W., New York (Academic Press).